# Supplementary material for: Evaluation of Eosinopenia as a SIRS Biomarker in Critically Ill Horses
Source: Animals (Basel). 2022 Dec 15;12(24):3547. doi: 10.3390/ani12243547 (PMC9774166; doi:10.3390/ani12243547)
Supplement: Supplementary file 1 [file animals-12-03547-s001.zip › animals-2048315-supplementary.pdf]

Supplementary Table S1. Demographics and clinical data of horses received in the emergency service on admission.

Horse	Group	Age (Years)	Gender	Heart rate (bpm)	Body Temperature (ºC)	Respiratory rate (bpm)
1	A	3	F	54	37.3	24
2	A	4	F	48	38.1	16
3	A	4	M	52	37.8	20
4	A	6	M	44	37.5	16
5	A	10	M	52	37.8	24
6	A	20	F	54	38.3	24
7	A	5	M	52	37.5	16
8	A	8	M	52	38.1	24
9	A	15	F	48	38.1	36
10	A	3	M	44	37.4	20
11	A	10	H	52	37.9	24
12	A	8	H	48	38.1	24
13	A	4	H	52	37.7	20
14	A	11	M	48	38.4	40
15	A	6	H	52	37.6	32
16	A	16	M	42	38.2	12
17	B	15	M	80	39.1	20
18	B	9	M	68	38.6	36
19	B	8	M	64	37.3	40
20	B	18	M	68	38.4	44
21	B	6	M	64	39.4	32
22	B	13	M	72	38.7	24
23	B	16	M	78	37.7	34
24	B	9	M	68	38.2	38
25	B	4	M	84	38.4	36
26	B	18	M	72	38.2	38
27	B	3	H	64	37.9	36
28	B	12	H	88	36.3	38
29	B	7	H	68	38.3	34
30	B	15	M	60	39.3	32
31	B	3	M	88	39.8	52
32	B	5	M	68	37.7	32
33	B	14	M	72	38.2	34
34	B	5	M	64	37.8	34
35	B	8	H	68	38.3	32
36	B	3	M	72	39.2	32
37	B	7	M	68	38.8	16

bpm: beats per minute; ºC: degree Celsius; rpm: respirations per minute; F: Female; M: Male;

Supplementary Table S2. Value of the differential WBC on admission.

Horse	Segmented Neutrophils (cell/ μL)	Band Neutrophils (cell/ μL)	Lymphocites (cell/ μL)	Monocytes (cell/ μL)
1	1296	0	3834	108
2	5457	0	4708	107
3	6586	0	2136	178
4	4662	0	1638	0
5	4928	0	1024	256
6	9516	0	2562	122
7	816	136	748	0
8	1452	0	1782	66
9	5916	91	2448	816
10	7521	109	1962	1308
11	4331	639	2130	0
12	3990	0	1254	399
13	156	0	4104	540
14	5184	0	2673	81
15	6552	0	2366	182
16	8710	0	3770	520
17	9370	0	1356	565
18	768	0	66	66
19	6840	0	760	0
20	9858	0	742	0
21	4928	968	2728	176
22	11136	384	1024	384
23	7347	837	930	186
24	5400	0	1440	288
25	1500	0	1200	300
26	3555	0	900	45
27	6370	0	2548	182
28	874	0	2812	38
29	7840	560	2688	0
30	13695	0	1550	0
31	1100	0	960	500
32	2508	0	3072	114
33	840	0	1260	0
34	1242	1064	1368	76
35	3250	0	1250	0
36	10360	1680	1960	140
37	6360	0	4240	0
